# Randomised controlled trials in pre-hospital trauma: a systematic mapping review

**DOI:** 10.1186/s13049-021-00880-8

**Published:** 2021-05-17

**Authors:** Matilda K. Björklund, Moira Cruickshank, Robbie A. Lendrum, Katie Gillies

**Affiliations:** 1 Health Services Research Unit, Health Sciences Building, Foresterhill, Aberdeen, UK; 2 NHS Lothian, Department of Anaesthesia, Critical Care and Pain Medicine, Royal Infirmary of Edinburgh, 51 Little France Crescent, Edinburgh, EH16 4SA UK; 3 Bart’s Health NHS Trust, Royal London Hospital, Whitechapel, London, E1 1BB UK; 4 London’s Air Ambulance, The Helipad, 17th Floor, Royal London Hospital, Whitechapel, London, E1 1BB UK

**Keywords:** Trauma, Injury, Prehospital, RCT, Mapping review

## Abstract

**Background:**

Trauma is a leading cause of morbidity and mortality worldwide with about 5.8 million deaths globally and the leading cause of death in those aged 45 and younger. The pre-hospital phase of traumatic injury is particularly important as care received during this phase has effects on survival. The need for high quality clinical trials in this area has been recognised for several years as a key priority to improve the evidence base and, ultimately, clinical care in prehospital trauma. We aimed to systematically map the existing evidence base for pre-hospital trauma trials, to identify knowledge gaps and inform decisions about the future research agenda.

**Methods:**

A systematic mapping review was conducted first employing a search of key databases (MEDLINE, CINAHL, EMBASE, and Cochrane Library from inception to March 23rd 2020) to identify randomised controlled trials within the pre-hospital trauma and injury setting. The evidence ‘map’ identified and described the characteristics of included studies and compared these studies against existing priorities for research. Narrative description of studies informed by analysis of relevant data using descriptive statistics was completed.

**Results:**

Twenty-three eligible studies, including 10,405 participants across 14 countries, were identified and included in the systematic map. No clear temporal or geographical trends in publications were identified. Studies were categorised into six broad categories based on intervention type with evaluations of fluid therapy and analgesia making up 60% of the included trials. Overall, studies were heterogenous with regard to individual interventions within categories and outcomes reported. There was poor reporting across several studies. No studies reported patient involvement in the design or conduct of the trials.

**Conclusion:**

This mapping review has highlighted that evidence from trials in prehospital trauma is sparse and where trials have been completed, the reporting is generally poor and study designs sub-optimal. There is a continued need, and significant scope, for improvement in a setting where high quality evidence has great potential to make a demonstrable impact on care and outcomes.

## Background

Trauma is a leading cause of morbidity and mortality worldwide with road traffic injuries and self-inflicted injuries being the most common causes of trauma [1]. According to the World Health Organisation (WHO), injuries cause 32% more deaths than malaria, tuberculosis, and HIV/AIDS combined, with about 5.8 million deaths globally being caused by injuries [2]. In the UK and the US major trauma is the leading cause of death in those under 45 years and a significant cause of short and long-term morbidity [3, 4]. The National Audit Office estimate that major trauma costs the NHS between £0.3 and £0.4 billion a year in immediate treatment with an annual lost economic output between £3.3 and £3.7 billion [3, 5]. Within a major trauma setting, the quality of care delivered to patients in a pre-hospital setting has a critical impact on their chances of survival and future quality of life [6, 7]. Despite the significant economic and health burdens associated with pre-hospital trauma there are still many unanswered questions about how to treat these patients effectively [8, 9].

Randomised controlled trials (RCTs) are key to informing best clinical practice. RCTs are a reliable way of assessing the effectiveness (both clinical and cost) of different healthcare interventions but are rare in pre-hospital trauma where studies are often based on registry data using prospective before-and-after designs [8]. This is partly because there are a number of significant logistical and methodological challenges to conducting RCTs in a pre-hospital trauma setting [10]. The major difficulties in conducting RCTs in pre-hospital trauma lie in various aspects of trial design and conduct. For example, a key consideration for trial design can relate to the sometimes small (and specialised) numbers of potentially eligible patients where a standard design might be difficult and therefore adaptive trial designs may be more appropriate [11]. From a conduct perspective, the importance of timeliness with regard to accessing the patient, randomisation (often remotely) and delivery of the intervention is also key. The broader ethical considerations relating to recruitment, capacity and consent under the challenging circumstances surrounding major trauma also need to be considered [12]. Futhermore, the complex nature of traumatic injuries can result in patients receiving multiple interventions (that may not be standardised in or between trauma centres) both pre and within hospital, making it very difficult to disentangle intervention effect on outcomes such as mortality [8].

Whilst RCTs in prehospital trauma care are challenging to design and deliver they are not impossible. Two existing reviews have collated the literature on prehospital trauma trials [13, 14]. One of these reviews, which aimed to develop a register of prehospital trauma RCTs, was published in 2002 and identified 24 studies published over 34 years. However, some of the interventions included are no longer in standard use and the information reported was limited (to intervention type, number of patients, and the adequacy of allocation concealment) [13]. The second review (conducted as part of a larger project to inform a trial) also identified a paucity of pre-hospital trauma RCTs, identifying 15 trials in 15 years, but was focused on interventions delivered by land-based ambulance services [14]. Given the tight focus of this previous work (e.g. minimal trial features reported, focused on land-based retrieval) an up to date review of published pre-hospital trauma trials is needed.

Systematic mapping reviews are a form of evidence synthesis that aim to conduct a systematic search of a broad and/or heterogeneous evidence base in order to plan or prioritise future evaluative syntheses and identify gaps in knowledge to inform future research needs [15]. This systematic mapping review aimed to identify RCTs in pre-hospital trauma settings and characterize the key components of these trials in terms of trial design and conduct and compare these trials to existing priorities for research in prehospital trauma. This review provides evidence to determine what research priorities remain unanswered and explore key considerations for future trial design and delivery.

## Methods

### Search strategy

To identify relevant literature for this review, four databases (MEDLINE, CINAHL, EMBASE, and Cochrane Library) were searched from inception to 23rd of March 2020. Title and abstract screening were limited to studies identified from 2000 onwards due to the identification of existing reviews that included the pre-2000 literature [13]. The key search words within titles and abstracts for identifying relevant studies were ‘prehospital’ or ‘pre-hospital’, EMS, HEMS, emergency, trauma*, injury*, trial and study. The strategy was largely informed by a previous scoping review and further developed in consultation with an Information Scientist and with input from a pre-hospital care clinician (RL) [14]. A full search strategy is available in Appendix.

### Eligibility criteria

Randomised controlled trials within the pre-hospital trauma and injury setting were considered eligible. Interventions had to target patients (rather than the medical personnel) and had to be delivered in the pre-hospital setting (at least partially i.e. intervention started in pre-hospital setting). Any comparators, any treatment duration and follow up periods were considered. Study outcomes had to be clinical or health related. Studies were excluded based on the following criteria: non-trauma pre-hospital trials (e.g. management of stroke or out of hospital medical cardiac arrest, etc.), intervention not delivered in a pre-hospital setting, intervention does not target the index trauma injury, studies conducted in low-middle income countries (as there are additional issues affecting the design and delivery of RCTS in these countries e.g. less developed pre-hospital services that are out with the scope of this protocol), studies published before the year 2000, and studies where no full report was available in English. Hypothetical trials and trials including healthy volunteers were also excluded.

### Eligibility screening process

Tiles and abstracts identified through the search were independently assessed against the eligibility criteria by two reviewers (MB and KG, each screening 50% of total) with a third reviewer (MC) screening a random 10% of the overall search output. Potentially eligible abstracts were further reviewed by the clinician reviewer (RL). Any disagreements regarding eligibility were discussed between the team to establish consensus. Full text articles were obtained for those studies that on initial screening were considered potentially relevant and were further assessed for inclusion. All full text papers were assessed independently by two reviewers (MB and KG) with disagreements resolved between the review team. Reference lists of all included studies were examined for further relevant studies.

### Data extraction

A data extraction form was developed by the review team and piloted on three eligible studies. Information from all included studies was extracted by one reviewer (MB) with a random sample of 25% of studies assessed independently by two other reviewers (KG and MC). The following summary data were extracted and summarised from each study; study setting; sample size, type of trauma, participant characteristics; intervention (i.e. content and delivery), comparator, outcome (i.e. primary outcome, measurement, timing); methodological considerations (including trial design including whether adaptive, number of arms, method of randomisation, consent process, and whether the trial was stopped early); overall result; and patient and public involvement.

### Data analysis

Data from the studies included in this review were analysed descriptively with descriptive statistics used for reporting frequencies where appropriate. Temporal and geographical data were represented in visual graphs to illustrate trends. All other data were presented in tabular form. Intervention category labels were agreed upon between all reviewers and informed by a previous review of pre-hospital trauma trials that categorised studies by intervention type [13]. Decisions relating to whether included trials were considered as adaptive designs was based on the information provided showing a pre-planned change that an adaptive design might permit as per guidance [16]. Meta-analysis was not appropriate due to the heterogeneity of the studies with regard to interventions and outcomes.

### Quality appraisal

Risk of bias was assessed using an abridged version of the Cochrane Risk of Bias Tool (version 1). Risk of bias was assessed using questions related to random sequence generation, allocation concealment, blinding of participants and personnel, and blinding of outcome assessment. The risk of bias was categorised as either ‘low’, ‘high’ or ‘unclear’. The assessment was carried out by one reviewer (MB) with a 25% random sample independently assessed by two other reviewers (KG and MC).

## Results

**Fig. 1:**
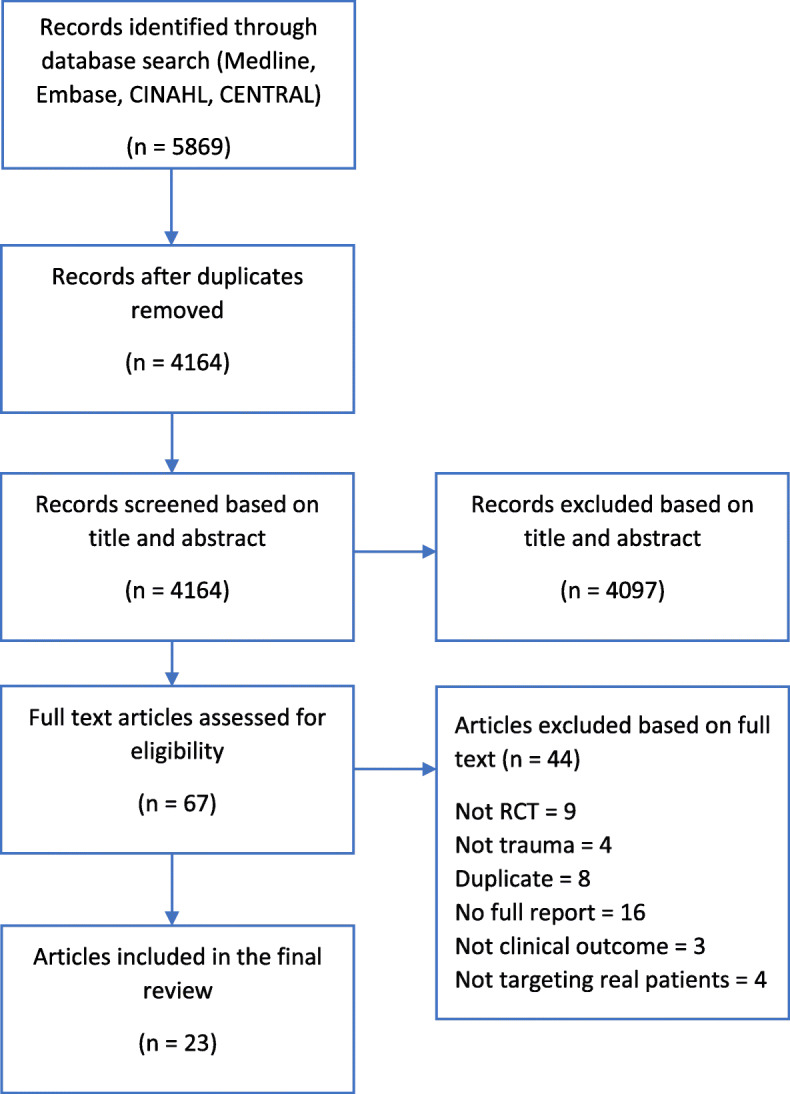
PRISMA diagram. RCT – Randomised controlled trial

After removing duplicates, 4164 unique references were identified from January 2000 to March 2020. The majority of these (*n* = 4097) were excluded as their title or abstract did not meet the eligibility criteria, which provided 67 articles for full text screening (see Fig. 1). A further 44 articles were excluded at full text screening resulting in a total of 23 studies being eligible and included in the mapping review [17–39].

### Study characteristics

**Fig. 2:**
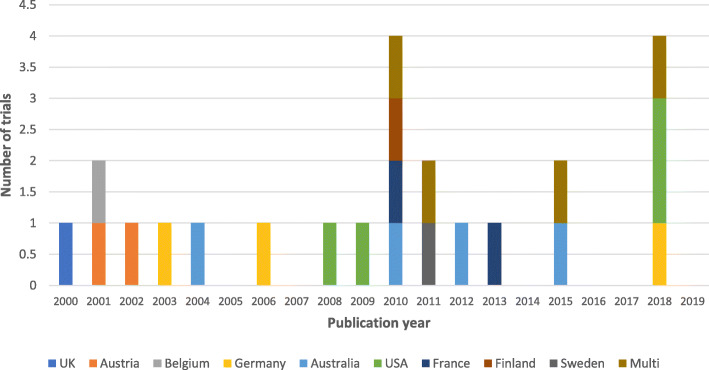
Geographical and temporal distribution of the included trials

The general study characteristics of all 23 studies can be seen in Table 1. There were slight differences in numbers of publication by year between 2000 and 2020 but no overall temporal trend (see Fig. 2). Most studies (*n* = 19, 82%) were conducted in single countries with Australia and the United States being the most common with four trials (17%) each (Fig. 2). Since 2010 there has been an increase in multi-national studies with four identified (18%), one of which involved two countries and the other involved six countries.

**Table 1:** Study characteristics

Study ID	Country	Sample size	Type of trauma	Intervention	Outcome of interest	Exemption from informed consent (EFIC) consultation reported
Turner 2000 [17]	England	1309	Penetrating and blunt trauma	Fluid therapy	Mortality	Not reported
Kober 2001 [34]	Austria	100	Minor trauma	Temperature management	Patient satisfaction; pain, discomfort and anxiety	Not reported
Vergnion 2001 [24]	Belgium	101	Not specified	Analgesia	Pain	Not reported
Kober 2002 [25]	Austria	60	Minor trauma	Analgesia	Pain and anxiety	Not reported
Helm 2003 [37]	Germany	97	Not specified	Airway management/ventilation	PaCO2 values as a marker of the adequacy of ventilation	Not reported
Cooper 2004 [18]	Australia	229	Blunt trauma	Fluid therapy	Recovery after brain injury	Not reported
ID27265 2006 [26]	Germany	136	Blunt trauma	Analgesia	Pain	Not reported
Bulger 2008 [19]	United States	209	Blunt trauma	Fluid therapy	Incidence of ARDS	Community notification and consultation were undertaken before the study.
Moore 2009 [31]	United States	714	Not specified	Blood product	Mortality	Exempt from informed consent stated - no consultation reported.
Bernard 2010 [38]	Australia	312	Penetrating and blunt trauma	Airway management/ventilation	Recovery after brain injury	Not reported
Bounes 2010 [27]	France	108	Not specified	Analgesia	Pain	Not reported
Bulger 2010 [20]	United States and Canada	1331	Blunt trauma	Fluid therapy	Recovery after brain injury	Community notification and consultation were undertaken before the study
Jousi 2010 [21]	Finland	37	Penetrating and blunt trauma	Fluid therapy	Blood pressure	Not reported
Bulger 2011 [22]	United States and Canada	895	Penetrating and blunt trauma	Fluid therapy	Mortality	Community notification and consultation were undertaken before the study
Lundgren 2011 [35]	Sweden	48	Blunt trauma	Temperature management	Body core temperature, cold discomfort and vital signs	Not reported
Jennings 2012 [28]	Australia	135	Not specified	Analgesia	Pain	Not reported
Ducasse 2013 [29]	France	60	Not specified	Analgesia	Pain	Not reported
Garner 2015 [39]	Australia	3124	Blunt trauma	Model of care	Recovery after brain injury	Not reported
Schreiber 2015 [23]	United States and Canada	192	Penetrating and blunt trauma	Fluid therapy	Early crystalloid volume; mortality	Exempt from informed consent stated - no consultation reported.
Büttner 2018 [30]	Germany	30	Not specified	Analgesia	Pain	Not reported
Cooper 2018 [36]	Australia, New Zealand, France, Switzerland, Saudi Arabia and Qatar	511	Not specified	Temperature management	Recovery after brain injury	Not reported
Moore 2018 [32]	United States	144	Not specified	Blood product	Mortality	Community notification and consultation were undertaken before the study
Sperry 2018 [33]	United States	523	Penetrating and blunt trauma	Blood product	Mortality	Community notification and consultation were undertaken before the study

*GOSE* Glasgow Outcome Scale Extended, *ARDS* Acute respiratory distress syndrome

The median number of participants in the studies was 144 (range 30-3124). Of the studies reporting type of trauma, both ‘blunt trauma’ and ‘blunt and penetrating trauma’ were the most frequently included populations accounting for six trials each and collectively more than half (*n* = 12, 52%) of the included studies. Nine studies did not specify whether the traumatic injury was blunt or traumatic and two studies included minor trauma patients. Public and patient involvement was not reported in any of the identified studies, but seven studies (all conducted in North America) included information on community consultation for exemption from informed consent [19, 20, 22, 23, 31–33]. Interventions were grouped into six broad categories: fluid therapy, analgesia, blood product, temperature management, airway management/ventilation, and model of care. These intervention categories were used to group and present the studies throughout the remainder of the review.

### Participant characteristics

Participant characteristics are summarised by intervention categories in Table 2. A total of 10,405 participants were included across the 23 trials included in this review. The mean age of participants in included trials was 42 years (SD 4.1) and the mean percentage of men was 60% (SD 23.4). In the three studies (13%) reporting ethnicity over 65% of participants were white [19, 31, 33]. Based on reported Injury Severity Scores (ISS measures trauma severity with a score of > 15 defining major trauma) the mean score was 21.9 (SD 8.7).

**Table 2:** Participant characteristics by intervention category

Intervention category	Age Mean (SD)	Sex % male	Ethnicity %	ISS Mean (SD)	Type of trauma
Fluid therapy (*n* = 7) [17–23]	38.3 (4.3)	72.0%	White 83% Asian 7% African American 5.5% American Indian 3% Pacific islander 0.5% Other/unknown 1% (Based on 1 study reporting data)	22.7 (9.9) (Based on 6 studies reporting data)	Penetrating and blunt trauma (Based on 5 studies reporting data)
Analgesia (*n* = 7) [24–30]	48.4 (13.3)	62.1%	Not reported	4 (Based on 1 study reporting data)	Blunt trauma (Based on 1 study reporting data)
Blood product (*n* = 3) [31–33]	37.8 (6.9)	77.9%	White 63% Black 24% Hispanic 9% Asian 1% Other 1% Unknown 1% (Based on 2 studies reporting data)	22.7 (3.8)	Penetrating and blunt trauma
Temperature management (*n* = 3) [34–36]	45.7 (12.0)	56.9%	Not reported	23 (Based on 1 study reporting data)	Blunt trauma (Based on 1 study reporting data)
Airway management/ventilation (*n* = 2) [37, 38]	38.4 (3.2)	73.1%	Not reported	31.7 (1.4)	Penetrating and blunt trauma (Based on 1 study reporting data)
Model of care (*n* = 1) [39]	43.5 (2.1)	73.5%	Not reported	27.5	Blunt trauma

*ISS* Injury Severity Score, *SD* Standard deviation

### Interventions and comparators

With regard to which interventions the RCTS were evaluating, more than half of the studies (*n* = 14, 60%) were evaluating fluid therapy or analgesia interventions (7 trials in each category). The remaining categories were populated as follows: blood product (*n* = 3), temperature management (*n* = 3), airway management/ventilation (*n* = 2), and model of care (*n* = 1) (see Table 3). The fluid therapy category, which includes 7 trials, had the highest total number of participants at 4202, followed by the model of care category at 3124 which only included one trial. Fluid therapy interventions (*n* = 7) were on the whole fairly homogeneous across studies and included intravenous fluid replacement protocols of various crystalloid/colloid solutions (normal saline, hypertonic saline, and hypertonic saline/colloid and Ringer’s lactate solution) with the exception of one study in which the intervention was an infusion of crystalloids and colloids. Analgesia interventions (*n* = 7) were heterogenous and included various forms of pain management, such as peripheral nerve blocks intravenous opioids or ketamine, nitrous oxide inhalation, topical diclofenac and acupressure. Blood product interventions included polymerised haemoglobin or thawed plasma. Temperature management interventions included external heating pads or blankets or induced mild hypothermia. Airway management and ventilation interventions included pre-hospital intubation by paramedics and monitor-adjusted ventilation by physicians. Only one study was categorised as a model of care in which the intervention evaluated dispatching a physician team in addition to a standard response team.

**Table 3:** Trial interventions and comparators by group

Intervention category	Total number of participants (median)	Interventions	Comparator	HCP delivering intervention	Transport mechanism
Fluid therapy (*n* = 7)	4202 (229)	- 500 mL of intravenous crystalloids and colloids (*n* = 1)	- Delayed or no infusion of intravenous fluids	- Paramedics	- Land based services
		- 250 mL intravenous infusion of 7.5% hypertonic saline and standard intravenous resuscitation fluids (crystalloids, Ringer’s lactate solution or colloids) (*n* = 1)	- 250 mL Ringer’s lactate solution and standard intravenous resuscitation fluids (crystalloids and colloids)	- Advanced life support paramedics	- Land based and air medical services
		- 250 mL hypertonic saline/colloid (7.5%/6%) or hypertonic saline (7.5%) (*n* = 2)	- Normal saline (0.9%) (*n* = 2)	- EMS personnel (*n* = 2)	- Land based and air medical services (*n* = 2)
		- 250 mL hypertonic saline/colloid (7.5%/6%) followed by Ringer’s lactate solution (non-racemic, L-lactate only) (*n* = 1)	- Conventional resuscitation and 250 mL Ringer’s lactate solution	- EMS personnel	- Land based and air medical services
		- 300 mL hypertonic saline 7.5% (*n* = 1)	- Conventional resuscitation (crystalloids and/or colloids)	- Emergency physicians	- Air medical services
		- 250 cc normal saline and 500 cc bottle of water (*n* = 1)	- 1000 cc normal saline and 2 l of fluid	- Advanced life support EMS	- Land based and air medical services
Analgesia (*n* = 7)	630 (101)	- 100 mg tramadol, more if required (up to 200 mg) (*n* = 1)	- 5 or 10 mg morphine, more if required	- Emergency physician	- Land based services
		- True acupressure; points Di4 (Hegu), KS9 (Schongchong), KS6 (Neiguan), BL60 (Kunlun), and LG20 (Baihui) (*n* = 1)	- False or no acupressure (*n* = 1)	- Paramedics	- Land based services
		- Diclofenac-ratiopharm Gel (1, 3% or 5%) 2-4 g twice daily (*n* = 1)	- Placebo gel	- Investigator and patient	- Land based services
		- Opioid titration protocol; intravenous 0.15 g/kg sufentanil followed by 0.075 g/kg until pain relief (*n* = 1)	- Intravenous 0.15 mg/kg morphine followed by 0.075 mg/kg until pain relief	- Physician, nurse, and EMT	- Land based services
		- Ketamine diluted in normal saline solution (10 mg/mL) after initial dose of morphine 5 mg intravenously (*n* = 1)	- Morphine diluted in normal saline solution (10 mg/9 mL) after initial dose of morphine 5 mg intravenously	- Paramedics	- Land based services
		- Pre-mixed 50% N_2_O and oxygen 9 L/min inhalation (*n* = 1)	- Medical air 9 L/min for 15 min followed by premixed 50% N_2_O and oxygen	- Nurse in Firefighter Emergency Services	- Land based services
		- Single shot peripheral block proximal to injury location under ultrasound guidance (*n* = 1)	- Intravenous analgesia using standard technique of s-ketamine combined with midazolam, in some cases fentanyl	- Emergency physicians	- Land based and air medical services
Blood product (*n* = 3)	1381 (523)	- Up to 6 U (50 g haemoglobin/unit) of PolyHeme from scene of injury and during first 12 h post-injury (*n* = 1)	- Crystalloids in the field and red blood cells as needed in hospital	- Paramedics	- Land based and air medical services
		- Thawed plasma (*n* = 2)	- Frozen water and normal saline (0.9%) as per standard care (*n* = 1) - Standard care resuscitation defined by local protocol (*n* = 1)	- Paramedics (*n* = 1) - Air medical personnel and physicians (*n* = 1)	- Land based services (*n* = 1) - Air medical services (*n* = 1)
Temperature management (*n* = 3)	659 (100)	- Resistive heating; carbon-fiber electric heating blanket set to 42 °C (*n* = 1)	- Passive warming	- Paramedics	- Land based services
		- A heating pad reaching 50 °C applied across anterior upper torso (*n* = 1)	- Polyester, woollen and rescue blankets	- EMS personnel	- Land based and air medical services
		- Prophylactic hypothermia by bolus of up to 2000 mL intravenous 4 °C saline (0.9%) and surface-cooling wraps targeting core temperature of 35 °C (*n* = 1)	- Normothermia; no exposure or cold fluids and warmed if required	- Emergency physicians	- Land based services
Airway management/ventilation (*n* = 2)	409 (204.5)	- Monitor; ventilation adjusted to achieve end-tidal carbon dioxide determined according to clinical condition of each patient, ventilation determined by weight of patients on admission (*n* = 1)	- Monitor-blind; ventilation set by using a tidal-volume of 10 mL kg^−1^ estimated body weight and age-appropriate ventilatory frequency	- Trauma anaesthesiologist and paramedic	- Air medical services
		- Pre-hospital intubation by paramedics (*n* = 1)	- In hospital intubation	- Intensive care paramedics	- Land based services
Model of care (*n* = 1)	3124 N/A	- Physician team in addition to standard response team (*n* = 1)	- Standard care by paramedics only	- Physicians and paramedics	- Air medical services

*HCP* Health Care Professional, *EMS* Emergency medical services, *EMT* Emergency medical technician

A range of comparators were evaluated in each of the intervention categories. Few studies specified what standard or conventional resuscitation protocols entailed or if they varied between study centres. In intervention categories where there was comparability between interventions (e.g. fluid therapy) often the comparators exhibited greater variability. Interventions were delivered by a range of personnel across the intervention categories and included either paramedics only (*n* = 6) or advanced life support paramedics/Emergency Medical Services (EMS) (*n* = 2), intensive care paramedic (*n* = 1), paramedics and other clinicians (trauma anaesthesiologists *n* = 1, physicians *n* = 1), EMS personnel (*n* = 4), emergency physicians (*n* = 4), a mix of providers (physician, nurse and emergency medical team *n* = 1, and air medical personnel and physicians *n* = 1); one study intervention was delivered by a nurse in a firefighter emergency services crew^,^ and one by the investigator and the patient. Similarly, to the range of individuals delivering the interventions, there was also variability in the transport mechanism of how medical teams attended the patients with 11 trials (48%) land-based only, eight trials land and air medical services, and four air medical services only. The analgesia intervention category again showing least variability with all but one of the studies being delivered through land-based services (See Table 3).

### Trial outcomes

The primary outcomes, measures, and time-point data from the included trials are summarised by intervention categories in Table 4. The primary outcomes in the fluid therapy intervention category exhibited significant heterogeneity, with 5 different outcomes across the 7 (largely similar) interventions being compared. These outcomes include mortality, morbidity, recovery after brain injury, incidence of acute respiratory distress syndrome (ARDS), early crystalloid volume, and blood pressure. As would be expected with this range of outcomes measured, the associated measurement tool and timing of outcome assessment also varied from admission to hospital, to 28 days after injury, and up to 6 months after injury. Analgesia intervention outcomes all reported pain, but how and when this was measured varied across studies. Studies reported the use of verbal rating scales, visual analogue scales, binary assessments of presence or absence of pain, or tenderness of area and outcome assessment timing varied from 15 min after intervention to arrival at hospital. The blood product interventions all measured mortality either at 28 or 30 days after injury. Temperature management intervention outcomes included recovery after brain injury, body core temperature, cold discomfort and vital signs, reduced thermal discomfort, pain, fear, and improved patient satisfaction. Airway management and ventilation intervention outcomes included recovery after brain injury and arterial blood gas PaCO2 values as a marker of the adequacy of ventilation. The variability in outcome selection in both the temperature management and airway management intervention groups was mirrored in the measurement tools and timing of outcome assessment. The most frequently reported primary outcomes were pain (*n* = 8) which was reported across 3 intervention categories in multiple different ways and time points, followed by mortality (*n* = 5) which was reported across 2 intervention categories with little variation in how and when, and recovery after brain injury (*n* = 5) reported across 4 intervention categories consistently using the Glasgow Outcome Score (or the extended version) at 6 months after injury. The retention rate (reported as proportion included in analysis) was higher in studies with primary outcomes of interest measured during a shorter time period.

**Table 4:** Trial outcomes

Intervention category	Primary outcome(s)	Measurement	Timing	Proportion included in analysis % Median (IQR)
Fluid therapy (*n* = 7)	- Mortality and morbidity (*n* = 1) - Survival (*n* = 1)	- Mortality – patients who died during follow up period (*n* = 1) - Morbidity – postal questionnaire; Short Form with 36 items (SF-36) (*n* = 1) - Survival (*n* = 1)	- 28 days after injury (*n* = 1) - 6 months after injury (*n* = 1)	98.6% (68.2 – 99.8)
	- Recovery after bran injury (*n* = 2)	- Glasgow Outcome Score – Extended; 8-point scale (*n* = 2)	- 6 months after injury (*n* = 2)	
	- Incidence of Acute respiratory distress syndrome (ARDS) (*n* = 1)	- Presence of ARDS; based on American-European Consensus Conference on ARDS definition	- 28 days after injury	
	- Early crystalloid volume (*n* = 1)	- Defined as crystalloid infused from EMS arrival until end of study period	- 28 days after injury	
	- Blood pressure (*n* = 1)	- Systolic blood pressure in mmHg	- Admission to hospital	
Analgesia (*n* = 7)	- Pain (*n* = 1)	- Pain intensity; verbal rating scale (0 none to 3 severe)	- 40 min after intervention	99.7% (96.3 – 100) (Based on 6 reporting studies)
	- Pain and anxiety (*n* = 1)	- 100-mm visual analogue scale (VAS) (0 no pain or no anxiety and 100 maximum pain or maximum anxiety)	- Arrival at hospital	
	- Pain (*n* = 1)	- Verbal numerical rating (0 no pain to 10 worst pain imaginable)	- Arrival at hospital	
	- Pain (*n* = 1)	- Tenderness measured by calibrated callipers in area of 1cm^2^ at the centre of injured area (marked on patient’s skin), measured between time of injury and 3 h thereafter	- 7 days after injury	
	- Pain (*n* = 2)	- Numeric rating scale (pain relief defined as 3 or lower of 10) (*n* = 2)	- 15 min after intervention (*n* = 1) - Arrival at hospital (*n* = 1)	
	- Pain (*n* = 1)	- Presence of pain; Yes or No	- 2 days after injury	
Blood product (*n* = 3)	- Mortality (*n* = 3)	- Mortality – patients who died during follow up period	- 28 days after injury (*n* = 1) - 30 days after injury (*n* = 2)	93.8% (N/A)
Temperature management (*n* = 3)	- Recovery after brain injury (*n* = 1)	- Glasgow Outcome Score – Extended; 8-point scale	- 6 months after injury	100% (N/A)
	- Body core temperature, cold discomfort and vital signs (*n* = 1)	- Temperature; closed ear canal temperature sensor (Smiths Medical, Ltd., UK) - Cold discomfort; numerical rating scale (0 no cold to 10 unbearable cold), very cold to hot - Vital signs; routine EMS equipment	- Arrival at hospital	
	- Improved patient satisfaction and reduced thermal discomfort, pain, and fear (*n* = 1)	- Satisfaction; rating scale (very good to unacceptable) - Pain; numerical rating scale (0 no pain to 5 worst imaginable pain) - Fear; rating scale (no, mild or severe anxiety)	- Arrival at hospital	
Airway management/ventilation (*n* = 2)	- Recovery after brain injury (*n* = 1)	- Glasgow Outcome Score – Extended; 8-point scale	- 6 months after injury	74.45% (N/A)
	- PaCO2 values as a marker of the adequacy of ventilation (*n* = 1)	- PaCO2 between 35 and 45 mmHg; (i-STAT, Hewlett Packard, Böblingen, Germany)	- Arrival at hospital	
Model of care (*n* = 1)	- Recovery after brain injury (*n* = 1)	- Glasgow Outcome Score; 5-point scale	- 6 months after injury	95.6%

### Methodological characteristics

The general methodological aspects of the studies were similar across the intervention categories (see Table 5). The included trials were generally poor at explicitly specifying the trial design with 19 (83%) not reporting the trial design used. Of the studies that did explicitly report design, three utilised a parallel design and one was a cluster trial design (with clusters defined as the ‘transporting base’). Given the challenges of potentially small samples in pre-hospital trauma trials we also investigated whether any of the included studies were adaptive designs. Seven of the included studies (30%) made explicit statements in their report that would indicate they were adaptive designs, a further 2 were unclear, and the remainder did not mention. The majority of the included studies (*n* = 20) were 2 arm trials with direct head-to head comparisons. Methods of randomisation varied across the included trials with the use of pre-randomised trial packs in seven of the studies, the use of sealed envelopes in a further seven, and other options such as telephone, toin coss or often not reported (*n* = 5). With regard to seeking informed consent, the majority of studies (*n* = 12, 52%) reported the use of a waiver of consent with consent sought for continued follow up, prospective verbal consent was sought in 7 studies (30%), one study reported consent was not required, and three studies did not report the consent model. Four studies were stopped early, two due to futility (fluid therapy and blood product) and two due to insufficient enrolment (fluid therapy and analgesia). Risk of bias assessment and evidence of effect are presented for each study in Table 6. Overall, random sequence generation was either ‘low’ or ‘unclear’ for all studies and a ‘high’ risk of bias was most frequently identified in blinding of participants and personnel, with most reported in the blood product and temperature management category. Evidence of effect (i.e. benefit from intervention) was found in 12 of the 23 studies (52%) and unclear in one (Table 6).

**Table 5:** Methodological considerations

Intervention category	Trial design	Adaptive design	Number of arms	Method of randomisation	Consent process	Early stopping
Fluid therapy (*n* = 7)	• Not reported (*n* = 7)	• Yes (*n* = 4) • Not reported (*n* = 3)	• 2 (*n* = 6) • 3 (*n* = 1)	• Pre-randomised study packs (*n* = 5) • Not reported (*n* = 2)	• Informed consent waived – consent for continued follow up (*n* = 5) • Not required (*n* = 1) • Not reported (*n* = 1)	- Futility (*n* = 1) - Insufficient enrolment (*n* = 1)
Analgesia (*n* = 7)	• Parallel (*n* = 3) • Not reported (*n* = 4)	• Not reported (*n* = 7)	• 2 (*n* = 5) • 3 (*n* = 1) • 4 (*n* = 1)	• Toss of a coin(*n* = 1) • Use of sealed opaque envelopes (*n* = 3) • Pre-randomised study packs (*n* = 1) • Not reported (*n* = 2)	• Informed consent waived – consent for continued follow up (*n* = 1) • Prospective verbal consent (*n* = 5) • Not reported (*n* = 1)	• Insufficient enrolment (*n* = 1)
Blood product (*n* = 3)	• Cluster (*n* = 1) • Not reported (*n* = 2)	• Yes (*n* = 1) • Unclear (*n* = 1) • Not reported (*n* = 1)	• 2 (*n* = 3)	• Service randomised (*n* = 1) • Pre-randomised study packs (*n* = 1) • Not reported (*n* = 1)	• Informed consent waived – consent for continued follow up (*n* = 3)	• Futility (*n* = 1)
Temperature management (=3)	• Not reported (*n* = 3)	• Yes (*n* = 1) • Not reported (*n* = 2)	• 2 (*n* = 3)	• Use of sealed opaque envelopes (*n* = 3)	• Prospective verbal consent (*n* = 2) • Informed consent waived – consent for continued follow up (*n* = 1)	• No early stopping
Airway management/ventilation (*n* = 2)	• Not reported (*n* = 2)	• Yes (*n* = 1) • Not reported (*n* = 1)	• 2 (*n* = 2)	• Use of sealed opaque envelopes (*n* = 1) • Not reported (*n* = 1)	• Informed consent waived – consent for continued follow up (*n* = 1) • Not reported (*n* = 1)	• No early stopping
Model of care (*n* = 1)	• Parallel (*n* = 1)	• Unclear (*n* = 1)	• 2 (*n* = 1)	• Centrally automated system	• Informed consent waived – consent for continued follow up (*n* = 1)	• No early stopping

**Table 6:**
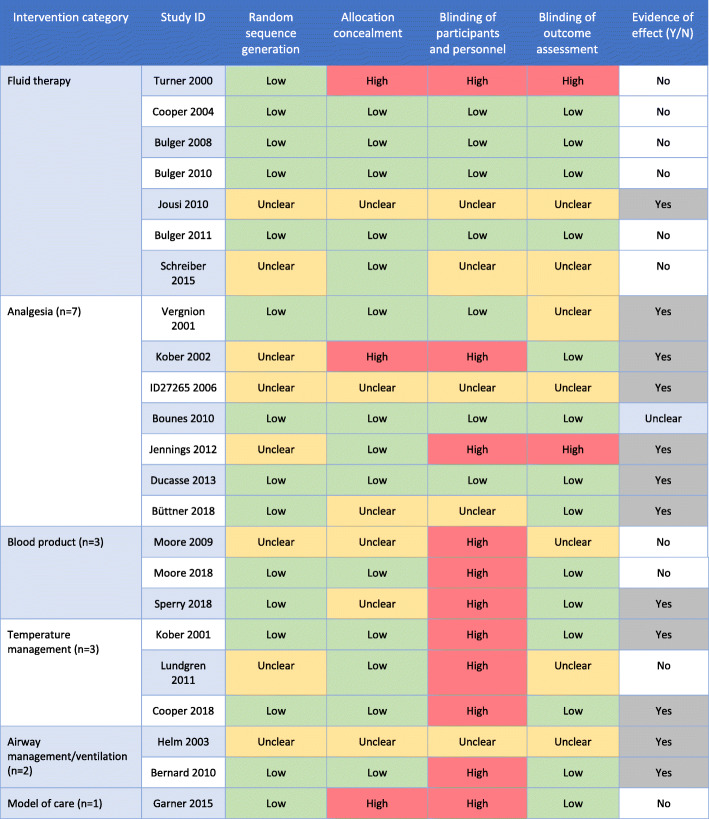
Risk of bias and Evidence of effect

Green = low risk of bias, Yellow = unclear risk of bias, Red = high risk of bias

### Research priorities

The evidence map was compared against European research priorities for pre-hospital critical care research (which includes trauma but is restricted to physician-provided care where as this review included trials regardless of practitioner) [9]. The evidence map presented in this review has identified randomised trials on eight (22%) of the 36 priorities, two of which feature in the top 5 priorities identified. These 8 priority areas include: Pre-hospital critical care -Staffing, training and effect (*n* = 1 trial identified); Advanced airway management in pre-hospital care (*n* = 2 trials); Pre-hospital temperature management in critical care patients (*n* = 3 trials); Monitoring in the pre-hospital setting (*n* = 1 trial); Fluid resuscitation in shock (*n* = 6 trials); Management of haemorrhagic shock (*n* = 3 trials); Pre-hospital analgesia-new perspectives (*n* = 7 trials); and Management of severe head injury (*n* = 5 trials). The mapping review identified 22 trials that contribute evidence to answering eight of these research priorities with some trials being considered across more than one priority. Fourteen (64%) of the trials, mapped to research priorities, were published pre-2011 before identification of the research priorities with eight (36%) published post 2011. Although this comparison of the identified evidence and the current priorities focuses on pre-hospital critical care in its entirety, it highlights the dearth of randomised evidence that exists for the other priorities including three of the top five which are all directly relevant to pre-hospital trauma care (see Table 7).

**Table 7:** Review findings mapped against research priorities for physician-provided pre-hospital critical care

No.	Suggested pre-hospital trauma research areas [9]	Evidence identified in mapping review
1	Pre-hospital critical care. Staffing, training and effect	✓
2	Advanced airway management in pre-hospital care	✓
3	Define time window for time-critical interventions	
4	Pre-hospital ultrasound	
5	Dispatch/activation criteria for physician-manned EMS	
6	Integrated information systems	
7	Evaluating quality of care	
8	Patient safety in the pre-hospital setting	
9	Pre-hospital temperature management in critical care patients	✓
10	Monitoring in the pre-hospital setting	✓
11	Fluid resuscitation in shock	✓
12	Efficient and reliable trauma registries	
13	Immobilization techniques	
14	Pre-hospital management of stroke	
15	Where to go with which patient?	
16	Emergency cardiac care in the pre-hospital setting	
17	Management of haemorrhagic shock	✓
18	Interhospital transport	
19	Does further centralization give better outcomes?	
20	Goal-directed therapy studies in pre-hospital critical care	
21	EMS systems - regionalization of emergency care	
22	Validity and impact of pre-hospital assessment	
23	Economic impact of EMS	
24	Pre-hospital analgesia, new perspectives	✓
25	Major incident management: How can it be improved?	
26	Management of severe head injury	✓
27	Pre-hospital recognition and goal-directed therapy of sepsis	
28	Paediatric transport solutions	
29	Implementation of new guidelines and research findings	
30	Effects of pre-hospital care on quality of life	
31	Ethical implications in pre-hospital research	
32	Pre-hospital care as a steering system for acute patients	
33	Lay person interventions before arrival of EMS	
34	Communication and interaction between EMS and hospitals	
35	Evaluation of future needs in pre-hospital care	
36	Pre-hospital thoracotomy	

## Discussion

This mapping review of the current evidence on pre-hospital trauma trials identified 23 studies published over the last 20 years. Of these 23 trials, 20 directly contribute evidence to research priorities for prehospital critical care (which includes major trauma but was focused on physician provided care). However, only six of these trials were published after the development of the prioritised list suggesting a more coordinated effort amongst the prehospital trauma trials community to develop trials that address the identified gaps in the evidence base. Overall, the mapping review has shown that there is significant heterogeneity within pre-hospital trauma trials, specifically in terms of interventions, comparators, and outcomes assessed. Whilst variation is expected to some extent due to the nature of the question driving design choices and interventions being investigated, the heterogeneity of comparators and outcomes being assessed where interventions are similar, creates problems for summarising intervention effects. This mapping review has identified significant gaps in the prehospital trauma evidence base when compared with current overlapping research priorities and has identified considerations for the future design and reporting of trials in this context.

The study populations of the included studies in this evidence map were fairly homogeneous with regard to key characteristics such as age and sex (as reported by included studies). Trauma and injury tends to affect men aged 45 and under more so than other demographic groups and our review found that the included studies reflected this (see Table 2) [3, 4]. The ethnicity of study participants was only reported by three studies, all of which were conducted in the United States (with one multi-national also including Canada), and reported the majority of participants to be white [19, 31, 33]. The fact that only three studies reported any data on participant ethnicity brings the validity of the representativeness of this data for the general trauma population into question. A 2019 systematic review of studies employing a community consultation survey on the exception from informed consent (EFIC), relevant for many pre-hospital trauma trials, found that the survey respondents were not an accurate representation of the trial population demographic with African American individuals underrepresented [40]. This supports the argument for further consideration of ethnicity as an important factor in trial design to ensure the study populations are truly representative of the population which they aim to serve. The lack of reporting of ethnicity of trial populations needs to be addressed.

Our review identified that fluid therapy and analgesia interventions made up 60% of the included studies. Fluid therapy interventions have been identified as a major intervention category in a previous review covering studies published pre-2002 but analgesic interventions only made up 12.5% of the trials identified previously [13]. Haemorrhage is the leading cause of potentially preventable death in trauma patients, especially in the pre-hospital and early stages [41]. Pre-hospital blood product transfusion may mitigate against this. However, the commonly accepted standard strategies of transfusion have been questioned [42]. Therefore, trials evaluating transfusion therapy interventions have remained a strong point of interest in pre-hospital trauma trials [43]. Yet despite interventions being similar the heterogeneity of outcomes in this category do not lend themselves to aggregative analyses and as such contribute to research waste. In addition, all of the interventions included in our evidence map related to haemorrhage were non-procedural, but there is now accumulating early evidence of benefit from the use of procedural interventions to treat trauma in a pre-hospital setting [44, 45]. Well designed (likely adaptive), multi-national, randomised comparisons of these advances in complex interventions, alongside complementary process evaluations to understand key aspects of trial delivery, are essential to improve the evidence base in this area.

The most common outcome reported in the included trials was pain, reported in eight (35%) trials, and patient-reported across a number of different tools and at different time points. Mortality and recovery after brain injury were the next most commonly reported outcomes in the included trials, both clinical outcomes with fairly standardised measures and timing of assessment. Other studies have also highlighted the heterogeneity of outcome measures in prehospital major trauma trials and recognised the importance of evaluating patient-centred outcomes in this area [46]. A systematic review and patient involvement exercise reported that major trauma survivors consider quality of life as the most important outcome for patients, followed by mortality or survival but this is contingent on longer term quality of life gains [46]. The problems of outcome heterogeneity and a lack of inclusion of patient-relevant outcomes in prehospital trauma trials could be addressed through the development and use of a suite of core outcome sets (an agreed standardised set of outcomes that should be measured and reported, as a minimum, in all clinical trials in specific areas of health or health care) [47].

The lack of reported patient and public involvement in the included trials points to the need for additional efforts in this area for pre-hospital trauma trials. In addition, the pre-hospital research prioritisation exercise takes into account the views of clinicians only and does not consider how those priorities might be different if patients had also been included [9]. Involving patients in this context can have additional challenges to some of the healthcare contexts but that should not exclude these important stakeholders from contributing to the research as active partners to ensure the evidence is fit for purpose. There are notable examples of how patients and the public are being actively involved, in prioritisation and the design and delivery of trials, in both emergency research and prehospital care trials [48–50]. Learning from these exemplars is critical to ensure that prehospital trauma trials produce evidence that is relevant and accountable [48–50].

Finally, the reporting of many of these studies with respect to key methodological considerations was poor across most included trials. Whilst not assessed in detail, even when considering what items are required to be reported in the CONSORT checklist, several of the included trials failed to report items such as: a clear description of trial design (such as parallel, factorial); randomisation sequence generation; and allocation concealment [51]. Given the often small sample sizes and requirements for responsive decision making based on accruing data there is likely potential utility for the use of adaptive trial designs for prehospital trauma trials. As such, attention to the recent adaptive design CONSORT extension will be key for those designing adaptive trials in this context to ensure adequate reporting [52]. Another consideration for trial design relates to the process of seeking consent in these pre-hospital trauma trials. Whist the majority of included trials had secured a waiver of consent, three studies did not report on the consent process and one stated consent was not required. Broader ethical questions and consultations with patients about appropriate mechanisms for consent in pre-hospital trauma trials is also required, in particular, around clinical trials of investigational medicinal (CTIMP) trials and non-CTIMP trials where the legislation across devolved nations in the UK is variable and as such makes running national trials in this context challenging.

### Strengths and limitations

This review was conducted in accordance with the general principles of the Cochrane Handbook for systematic reviews of interventions as adapted for a mapping review. One of the strengths of this review is the robustness of the methods used through double screening at every stage of the review process. Moreover, the search strategy was comprehensive and covered multiple databases.

However, there was a trade off in ensuring inclusion of relevant studies such that we did not define ‘major trauma’ in a prehospital setting and as such two prehospital minor trauma trials ended up being included in the review [25, 34]. This review limited study inclusion and studies conducted in low-middle income countries and trials not published in English were excluded. However, this resulted in the exclusion of only four studies of which one was conducted in a low-middle income country. Finally, the infrequent reporting of various study aspects relating to participant characteristics, interventions, outcomes, and study measures likely affects the overall generalisability of the results as they may be skewed. Risk of bias assessment especially was affected by unclear reporting of blinding measures in many of the studies which limits the inferences that can be made regarding the quality of the methodological aspects of the studies. Much of these limitations can be addressed by improving reporting of future pre-hospital trauma trials. It is important to highlight that whilst the findings from the mapping review were compared against an existing set of priorities for pre-hospital research, there are some important differences between the scope of the prioritisation exercise and this review. Namely, the prioritisation exercise considered physician only provided care (where we included trials regardless of practitioner), in prehospital critical care (which could include trauma but is not limited to), and the aim of the prioritisation was to identify the top 5 (where as we compare our findings across all identified priorities).

Finally, since the completion of the search in March 2020 and the publication of this review, a handful of trials which would be eligible for inclusion have been published. These trials have focused on interventions targeting haemorrhage management such as tranexamic acid [53], fibrinogen concentrate [54], and pelvic splinting to minimise bleeding [55]. Whilst important trials in this field, as is they would not change the overall findings of this mapping review. However, it will be important to include them in any future update of this review.

## Conclusions

This review has successfully identified studies and mapped the evidence for pre-hospital trauma trials published over the last 20 years. This evidence map, when considered alongside recent prioritisation exercises, can lend itself as a strong basis for informing future trials in pre-hospital trauma both in terms of what to start doing, what to keep doing and what to do less of. Identifying the gaps in the research, especially in their reporting and lack of involvement of patients as partners, is important for highlighting the areas for future research to improve upon. Ultimately this review highlights the need (and scope) for a paradigm shift in the design and conduct of RCTs in this setting. There is a critical need for the delivery of well-designed and executed RCTs in prehospital trauma to generate high quality evidence and guide clinical practice.

## Data Availability

No further data are available. All studies included in the review and are listed in the reference section. No additional data was sought from authors.

## Appendix

### Search Strategy

**Database: Ovid MEDLINE(R) and Epub Ahead of Print, In-Process & Other Non-Indexed Citations, Daily and Versions(R) < 1946 to March 23, 2020>.**

1 Emergency Medical Services/.

2 (pre-hospital or prehospital or “out of hospital” or ambulance or paramedic? or EMS).tw,kw.

3 1 or 2.

4 exp. “Wounds and Injuries”/.

5 (trauma or wound*1 or injur* or h?emorrhag*).tw,kw.

6 4 or 5.

7 (randomized controlled trial or controlled clinical trial).pt.

8 ((controlled or clinical or randomi#ed. or feasibility or pilot) adj2 (trial or study)).tw,kw.

9 7 or 8.

10 3 and 6 and 9.

**Database: Embase < 1974 to 2020 Week 13>.**

1 exp. emergency health service/.

2 (pre-hospital or prehospital or “out of hospital” or ambulance or paramedic? or EMS).tw,kw.

3 1 or 2.

4 exp. injury/.

5 (trauma or wound*1 or injur* or h?emorrhag*).tw,kw.

6 4 or 5.

7 ((controlled or clinical or randomi#ed. or feasibility or pilot) adj2 (trial or study)).tw,kw.

8 exp. controlled clinical trial/.

9 7 or 8.

10 3 and 6 and 9.

**CENTRAL.**

ID Search.

#1 MeSH descriptor: [Emergency Medical Services] explode all trees.

#2 (pre-hospital or prehospital or “out of hospital” or ambulance or paramedic? or EMS).

#3 #1 or #2.

#4 MeSH descriptor: [Wounds and Injuries] explode all trees.

#5 (trauma or wound*1 or injur* or h?emorrhag*).

#6 #4 or #5.

#7 #3 and #6.

**CINAHL.**

S1 (MM “Emergency Medical Services+”).

S2 (MM “Emergency Medical Technicians”).

S3 TX pre-hospital or prehospital or “out of hospital” or ambulance or paramedic? or EMS.

S4 TX s1 or s2 or s3.

S5 (MM “Trauma+”) OR (MM “Wounds and Injuries+”).

S6 TX (trauma or wound*1 or injur* or h?emorrhag*).

S7 s5 or s6.

S8 TX (controlled or clinical or randomi#ed. or feasibility or pilot) N2 (trial or study).

S9 s4 and s7 and s8.

## References

[1] Varghese MS, Kellermann A, Lormand JD (2005). Prehospital trauma care systems.

[2] World Health Organization (2010). Injuries and violence.

[3] National Institute for Health and Care Excellence (2016). Major trauma: assessment and initial management (NICE guideline NG39).

[4] Rhee P, Joseph B, Pandit V, Aziz H, Vercruysse G, Kulvatunyou N (2014). Increasing trauma deaths in the United States. Ann Surg.

[5] National Audit Office (2010). Major trauma Care in England.

[6] Hussain LM, Redmond AD (1994). Are pre- hospital deaths from accidental injury preventable?. BMJ.

[7] Patton GC, Coffey C, Sawyer SM (2009). Global patterns of mortality in young people: a systematic analysis of population health data. Lancet.

[8] Lockey DJ (2017). Research questions in pre-hospital trauma care. PLoS Med.

[9] Fevang E, Lockey D, Thompson J (2011). The top five research priorities in physician-provided pre-hospital critical care: a consensus report from a European research collaboration. Scand J Trauma Resusc Emerg Med.

[10] Maurin Söderholm H, Andersson H, Andersson Hagiwara M, Backlund P, Bergman J, Lundberg L (2019). Research challenges in prehospital care: the need for a simulation-based prehospital research laboratory. Adv Simul.

[11] Jansen J, Pallmann P, MacLennan G, Campbell MK (2017). UK-REBOA trial investigators. Bayesian clinical trial designs: another option for trauma trials? [published correction appears in J trauma acute care Surg. 2019 Apr;86(4):760]. J Trauma Acute Care Surg.

[12] Armstrong S, Langlois A, Siriwardena N, Quinn T (2019). Ethical considerations in prehospital ambulance based research: qualitative interview study of expert informants. BMC Med Ethics.

[13] Kwan I, Bunn F, Roberts I, Wentz R (2002). The development of a register of randomized controlled trials in prehospital trauma care. Prehos Emerg Care.

[14] Lecky F, Russell W, Fuller G, McClelland G, Pennington E, Goodacre S (2016). The head injury transportation straight to neurosurgery (HITS-NS) randomised trial: a feasibility study. Health Technol Assess.

[15] James KL, Randall NP, Haddaway NR (2016). A methodology for systematic mapping in environmental sciences. Environ Evid.

[16] Pallmann P, Bedding AW, Choodari-Oskooei B, Dimairo M, Flight L, Hampson LV, Holmes J, Mander AP, Odondi L, Sydes MR, Villar SS, Wason JMS, Weir CJ, Wheeler GM, Yap C, Jaki T (2018). Adaptive designs in clinical trials: why use them, and how to run and report them. BMC Med.

[17] Turner J, Nicholl J, Webber L, Cox H, Dixon S, Yates D (2000). A randomised controlled trial of prehospital intravenous fluid replacement therapy in serious trauma. Health Technol Assess.

[18] Cooper D, Myles P, McDermott F, Murray L, Laidlaw J, Cooper G (2004). Prehospital hypertonic saline resuscitation of patients with hypotension and severe traumatic brain injury. JAMA..

[19] Bulger E, Jurkovich G, Nathens A, Copass M, Hanson S, Cooper C (2008). Hypertonic resuscitation of hypovolemic shock after blunt trauma. Arch Surg.

[20] Bulger E, May S, Brasel K, Schreiber M, Kerby J, Tisherman S (2010). Out-of-hospital hypertonic resuscitation following severe traumatic brain injury. JAMA..

[21] Jousi M, Reitala J, Lund V, Katila A, Leppäniemi A (2010). The role of pre-hospital blood gas analysis in trauma resuscitation. World J Emerg Surg.

[22] Bulger E, May S, Kerby J, Emerson S, Stiell I, Schreiber M (2011). Out-of-hospital hypertonic resuscitation after traumatic hypovolemic shock. Ann Surg.

[23] Schreiber M, Meier E, Tisherman S, Kerby J, Newgard C, Brasel K (2015). A controlled resuscitation strategy is feasible and safe in hypotensive trauma patients. J Trauma Acute Care Surg.

[24] Vergnion M, Degesves S, Garcet L, Magotteaux V (2001). Tramadol, an alternative to morphine for treating posttraumatic pain in the Prehospital situation. Anesth Analg.

[25] Kober A, Scheck T, Greher M, Lieba F, Fleischhackl R, Fleischhackl S (2002). Prehospital analgesia with acupressure in victims of minor trauma: a prospective, randomized, double-blinded trial. Anesth Analg.

[26] European Union Clinical Trials Register. Germany: database publisher. 29 May 2006 - . Identifier EUCTR2005-000888-26-DE, Randomised, double-blind, multi-centre, placebo-controlled clinical dose finding study in four parallel groups comparing Diclofenac Gel 1%, Diclofenac Gel 3%, Diclofenac Gel 5% and placebo Gel in patients with traumatic blunt soft tissue injury/contusion; 2006 May 29 [7 pages]. Available from: https://www.clinicaltrialsregister.eu/ctr-search/trial/2005-000888-26/results. Cited 2020 July 20.

[27] Bounes V, Barthélémy R, Diez O, Charpentier S, Montastruc J, Ducassé J (2010). Sufentanil is not superior to morphine for the treatment of acute traumatic pain in an emergency setting: a randomized, double-blind, out-of-hospital trial. Ann Emerg Med.

[28] Jennings P, Cameron P, Bernard S, Walker T, Jolley D, Fitzgerald M (2012). Morphine and ketamine is superior to morphine alone for out-of-hospital trauma analgesia: a randomized controlled trial. Ann Emerg Med.

[29] Ducassé J, Siksik G, Durand-Béchu M, Couarraze S, Vallé B, Lecoules N (2013). Nitrous oxide for Early analgesia in the emergency setting: a randomized, double-blind multicenter Prehospital trial. Acad Emerg Med.

[30] Büttner B, Mansur A, Kalmbach M, Hinz J, Volk T, Szalai K (2018). Prehospital ultrasound-guided nerve blocks improve reduction-feasibility of dislocated extremity injuries compared to systemic analgesia. A randomized controlled trial. PLoS One.

[31] Moore E, Moore F, Fabian T, Bernard A, Fulda G, Hoyt D (2009). Human polymerized hemoglobin for the treatment of hemorrhagic shock when blood is unavailable: the USA multicenter trial. J Am Coll Surg.

[32] Moore H, Moore E, Chapman M, McVaney K, Bryskiewicz G, Blechar R (2018). Plasma-first resuscitation to treat haemorrhagic shock during emergency ground transportation in an urban area: a randomised trial. Lancet.

[33] Sperry J, Guyette F, Brown J, Yazer M, Triulzi D, Early-Young B (2018). Prehospital plasma during air medical transport in trauma patients at risk for hemorrhagic shock. N Engl J Med.

[34] Kober A, Scheck T, FüLesdi B, Lieba F, Vlach W, Friedman A (2001). Effectiveness of resistive heating compared with passive warming in treating hypothermia associated with minor trauma: a randomized trial. Mayo Clin Proc.

[35] Lundgren P, Henriksson O, Naredi P, Björnstig U (2011). The effect of active warming in prehospital trauma care during road and air ambulance transportation - a clinical randomized trial. Scand J Trauma Resusc Emerg Med.

[36] Cooper D, Nichol A, Bailey M, Bernard S, Cameron P, Pili-Floury S (2018). Effect of Early sustained prophylactic hypothermia on neurologic outcomes among patients with severe traumatic brain injury. JAMA..

[37] Helm M, Schuster R, Hauke J, Lampl L (2003). Tight control of prehospital ventilation by capnography in major trauma victims. Br J Anaesth.

[38] Bernard S, Nguyen V, Cameron P, Masci K, Fitzgerald M, Cooper D (2010). Prehospital rapid sequence intubation improves functional outcome for patients with severe traumatic brain injury. Ann Surg.

[39] Garner AA, Mann KP, Fearnside M (2015). The head injury retrieval trial (HIRT): a single-Centre randomised controlled trial of physician prehospital management of severe blunt head injury compared with management by paramedics only. Emerg Med J.

[40] Feldman W, Hey S, Franklin J, Kesselheim A (2019). Public approval of exception from informed consent in emergency clinical trials. JAMA Netw Open.

[41] Callcut R, Kornblith L, Conroy A (2019). The why and how our trauma patients die. J Trauma Acute Care Surg.

[42] Chatrath V, Khetarpal R, Ahuja J (2015). Fluid management in patients with trauma: restrictive versus liberal approach. J Anaesthesiol Clin Pharmacol.

[43] 10.1186/ISRCTN14998314 Last accessed 21 Oct 20.

[44] Lendrum R, Perkins Z, Chana M, Marsden M, Davenport R, Grier G (2019). Pre-hospital resuscitative endovascular balloon occlusion of the aorta (REBOA) for exsanguinating pelvic haemorrhage. Resuscitation.

[45] Rago AP, Larentzakis A, Marini J, Picard A, Duggan MJ, Busold R, Helmick M, Zugates G, Beagle J, Sharma U, King DR (2015). Efficacy of a prehospital self-expanding polyurethane foam for noncompressible hemorrhage under extreme operational conditions. J Trauma Acute Care Surg.

[46] Hancox J, Toman E, Brace-McDonnell S, Naumann D (2019). Patient-centred outcomes for prehospital trauma trials: a systematic review and patient involvement exercise. Trauma..

[47] Williamson PR, Altman DG, Bagley H (2017). The COMET handbook: version 1.0. Trials.

[48] Evans B, Bulger J, Ford S, et al. Public and patient involvement in prehospital care research development – designing the rapid 2 trial. BMJ Open. 2019;9. 10.1136/bmjopen-2019-EMS.22.

[49] Hirst E, Irving A, Goodacre S (2016). Patient and public involvement in emergency care research. Emerg Med J.

[50] https://www.jla.nihr.ac.uk/priority-setting-partnerships/emergency-medicine/top-10-priorities.htm Last accessed 21 Oct 2020.

[51] Schulz KF, Altman DG, Moher D, for the CONSORT Group (2010). CONSORT 2010 statement: updated guidelines for reporting parallel group randomised trials. BMJ.

[52] Munyaradzi D, Philip P, James W, Susan T, Thomas J, Julious Steven A (2020). The adaptive designs CONSORT extension (ACE) statement: a checklist with explanation and elaboration guideline for reporting randomised trials that use an adaptive design. BMJ.

[53] Guyette FX, Brown JB, Zenati MS, Early-Young BJ, Adams PW, Eastridge BJ, Nirula R, Vercruysse GA, O'Keeffe T, Joseph B, Alarcon LH, Callaway CW, Zuckerbraun BS, Neal MD, Forsythe RM, Rosengart MR, Billiar TR, Yealy DM, Peitzman AB, Sperry JL, STAAMP Study Group (2020). Tranexamic acid during prehospital transport in patients at risk for hemorrhage after injury: a double-blind, placebo-controlled, randomized clinical trial. JAMA Surg.

[54] Ziegler B, Bachler M, Haberfellner H, Niederwanger C, Innerhofer P, Hell T, Kaufmann M, Maegele M, Martinowitz U, Nebl C, Oswald E, Schöchl H, Schenk B, Thaler M, Treichl B, Voelckel W, Zykova I, Wimmer C, Fries D, FIinTIC study group (2021). Efficacy of prehospital administration of fibrinogen concentrate in trauma patients bleeding or presumed to bleed (FIinTIC): a multicentre, double-blind, placebo-controlled, randomised pilot study. Eur J Anaesthesiol.

[55] Pierrie SN, Seymour RB, Wally MK, Studnek J, Infinger A, Hsu JR (2021). Evidence-based musculoskeletal injury and trauma collaborative (EMIT). Pilot randomized trial of pre-hospital advanced therapies for the control of hemorrhage (PATCH) using pelvic binders. Am J Emerg Med.

